# Safety and efficacy of hysteroscopic resection of uterine leiomyoma embedded at the base of a uterine septum

**Published:** 2021-01-08

**Authors:** O Abuzeid, M Ibrahim, S Joseph, J Herbert, M Abuzeid

**Affiliations:** Fellow, Division of Maternal Fetal-Medicine, Department of Obstetrics and Gynecology, Stony Brook University, Long Island, New York, USA; IVF Michigan Rochester Hills and Flint PC, Rochester Hills, Michigan, USA; Department of Obstetrics and Gynecology, Hurley Medical Center, Michigan State University, College of Human Medicine, Flint, Michigan, USA; Division of Reproductive Endocrinology and Infertility, Department of Obstetrics and Gynecology, Hurley Medical Center, Michigan State University, College of Human Medicine, Flint, Michigan, USA.

**Keywords:** Fibroid, Hysteroscopy, Myomectomy, Uterine Septum

## Abstract

**Background:**

To determine the safety and efficacy of hysteroscopic resection of uterine leiomyoma embedded at the base of a uterine septum

**Methods:**

This case series included 11 patients with infertility or recurrent pregnancy loss who were found to have a uterine septum (one septate and 10 sub-septate) and a uterine leiomyoma embedded at the base of the uterine septum. All patients underwent a hysteroscopic division of the uterine septum and hysteroscopic resection of the uterine leiomyoma. Safety was determined by any intra-operative complications, and any immediate or late postoperative complications. Efficacy was determined based on the findings on a postoperative trans-vaginal 3D ultrasound (TV 3D US) with a saline infusion sonohysterogram (SIH) and reproductive outcomes.

**Results:**

There were no reported intra-operative complications, or immediate or late postoperative complications. Eleven patients underwent TV 3D US with SIH; findings were normal in 8 (72.7%); 3 patients underwent a second operative hysteroscopy and subsequent TV 3D US with SIH were also normal. The analysis of reproductive outcomes was limited to patients who were < 40 years (9 patients). Seven patients conceived (77.8%), six delivered (66.7%) and one miscarried (14.3%).

**Conclusions:**

Hysteroscopic myomectomy of a leiomyoma, which is embedded at the base of a uterine septum, can be safely performed at the same session of hysteroscopic division of the uterine anomaly. Improvement in reproductive outcomes is to be expected after such procedures.

## Introduction

The presence of submucosal leiomyomas, that alter the uterine cavity, have been shown to increase the chances of reproductive failure (Pritts et al., 2009; Orisaka et al., 2007). Uterine anomalies, especially uterine septum, have been associated with pregnancy complications including miscarriages, preterm deliveries, fetal malpresentation, retained placentas and uterine ruptures (Braun et al., 2005; Grimbizis et al., 2001; Heinonen et al., 1982; Lin 2004; Rackow and Arici 2007; Saravelos et al., 2008; Troiano and McCarthy 2004; Woelfer et al., 2001; Zlopasa et al., 2007; Daly et al., 1989). Uterine septum is the most common anomaly accounting for about 55% of congenital Müllerian anomaly (Acien 1993). A successful hysteroscopic resection of submucosal leiomyomas can enhance reproductive outcomes (Fernandez et al., 2001; Jayakrishnan et al., 2013). In a recent review and meta-analysis, Valle and Ekpo (2013) reported a viable pregnancy rate of 80% after hysteroscopic metroplasty in patients with a septate uterus. Theoretically, some patients presenting with infertility, recurrent pregnancy loss and a bad obstetric history may have co-existing uterine septa and leiomyomas. There are only five separate case reports of such association in English literature (Caliskan et al., 2008; Iwase et al., 2013; Javaid et al., 2013; Abuzeid et al., 2017a; Abuzeid et al., 2017b). In this case series, we discuss the diagnosis and management of 11 infertile patients, who were found to have a rare combination of a uterine septum and a uterine fibroid embedded in its base. The purpose of this study is to determine the safety and efficacy of concomitant hysteroscopic division of uterine septum and hysteroscopic resection of uterine fibroid embedded at the base of the uterine anomaly at the same setting.

## Materials and methods

This retrospective case study included 11 patients who underwent a concomitant hysteroscopic correction of a uterine septum and uterine fibroids embedded at its base at the same setting. The study was conducted between 2015 to 2018, and it received an exemption from the oversight of the Hurley Medical Center Institutional Review Board (IRB). All patients presented with infertility and two patients presented with both infertility and recurrent pregnancy loss (RPL) [18.2%]. Work-up of infertility included: complete semen analysis, hysterosalpingogram (HSG), trans-vaginal (TV) 2D ultrasound scan (US), TV 3D US, TV 3D US with a saline infusion sonohysterogram (SIH), hormonal profile including serum TSH, prolactin, day 3 FSH and LH levels, anti-Müllerian hormone and laparoscopy and hysteroscopy when indicated. For patients with RPL, work-up to rule out endocrine factors, antiphospholipid antibody syndrome, abnormal chromosomes in male and female, and uterine factors was carried out.

**Figure 1:**
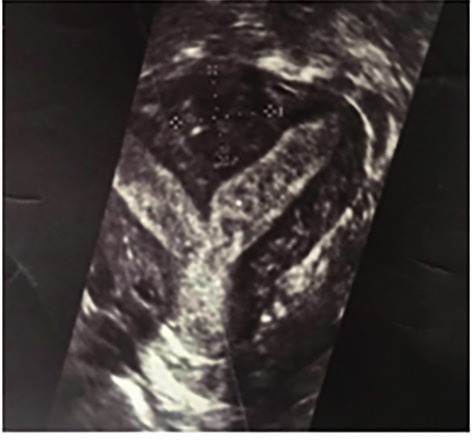
TV 3D US picture of a complete uterine septum with a fibroid embedded at its base in the fundal area of the uterus.

Any fibroid detected on TV 2D US or TV 3D US with or without SIH was described with respect to its type, location and size (Figure 1). Whenever a uterine septum was detected, it was classified according to the new classification of Müllerian anomalies proposed by the European Society of Human Reproduction and Embryology and the European Society for Gynecological Endoscopy (ESHRE-ESGE) (Grimbizis et al., 2013). In this classification, the definition of septate uterus is made if the internal indentation length of the fundal midline (IILFM) is > 50% of myometrial wall thickness in the fundal region (Grimbizis et al., 2013). For the purpose of this study, a diagnosis of a complete septate uterus (CSU) was made if the central point of indentation was at an acute angle (<90°), and reached the region of the internal or external cervical os (Figure 1). For the diagnosis of a partial septate uterus (PSU) we used a modification of ESHRE-ESGE in terms of the length of the IILFM as proposed by the Congenital Uterine Malformation by Experts (CUME) [Ludwin et al., 2018]. Therefore, a diagnosis of a partial septate uterus (PSU) was made if the central point of indentation was at an acute angle (<90°) [PSUAA] or at an obtuse angle (>90°) [PSUOA] and if the IILFM was > 10 mm (Ludwin et al., 2018).

**Figure 2:**
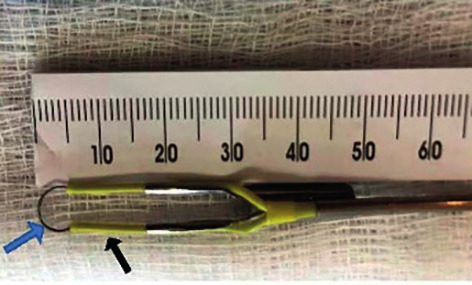
This picture illustrates the length of various parts of the straight resectoscope loop electrode of the ACMI hysteroscope that was used to measure IILFM and the size of any uterine fibroids embedded at the base of the uterine septum. The black cutting metal wire of the straight resectoscope loop electrode is 5mm in length (blue arrow), while the yellow insulating portion of the resectoscope loop is 10mm in length (black arrow).

Hysteroscopy was performed under modified general anaesthesia at the mid-follicular phase of the menstrual cycle, or while the patient was on oral contraceptives. Medications commonly used included Propofol, Versed and Fentanyl. Hysteroscopy combined with a laparoscopic procedure was performed under general endotracheal anaesthesia. An ACMI hysteroscope (Division of Olympus; Maple Grove, MN, USA) was used in all patients. Glycine 1.5% was used as a distension medium. During hysteroscopy the IILFM or the size of the fibroid seen embedded at the base of the septum was measured by comparing its length to the length of the cutting wire and the yellow insulating portion of the straight resectoscope loop electrode of the ACMI hysteroscopic resectoscope (Division of Olympus; Maple Grove, MN, USA) (Figure 2) (Abuzeid et al., 2014). The straight resectoscope loop electrode was used to divide the uterine septum in its mid portion, utilizing a monopolar system. A zero degree or 12-degree hysteroscope was used. The distension medium was Glycine 1.5%. A cutting current of 70 watts and a coagulation current of 50 watts with a blend of 1 was used.

**Figure 3:**
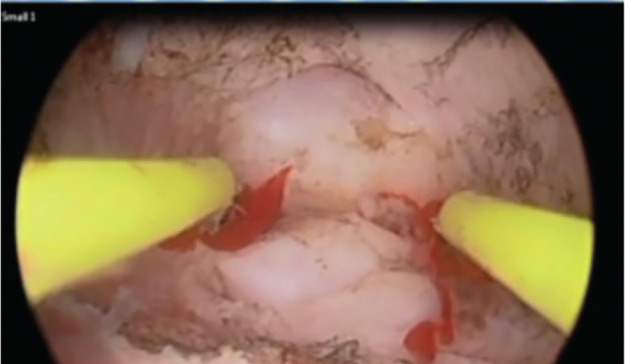
This picture illustrates the use of a straight resectoscope loop to slice the fibroid that was embedded at the base of the divided uterine septum into 2 pieces.

**Figure 4:**
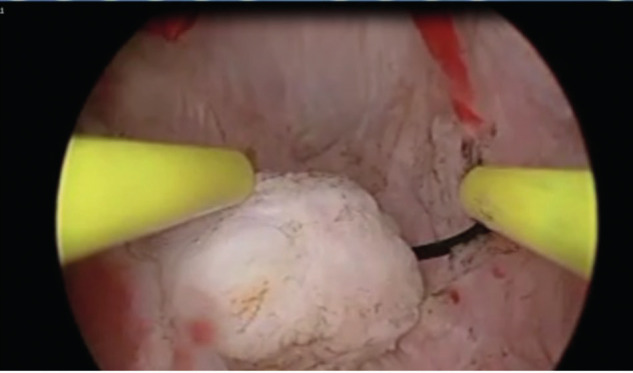
This picture illustrates the use of a straight resectoscope loop to re-sect one portion of the fibroid which was sliced into 2 pieces in Figure 3.

The procedure was terminated when we were able to move the tip straight resectoscope loop electrode from the area of one tubal ostium to the other without any resistance, achieving a triangular and symmetrical uterine cavity. Once the uterine fibroid that was embedded at the base of the uterine anomaly was visualized, a hysteroscopic myomectomy was performed in a routine fashion as described before (Abuzeid et al., 2017a; Abuzeid et al., 2017b; Abuzeid and Joseph 2014). In short, the straight resectoscope loop was used to slice the fibroid into 2 pieces (Figure 3). Thereafter, a straight or a right angled resectoscope loop was utilized to resect the fibroid free from the base of the septum (Figure 4). In one patient, (patient # 11) who was found to have a PSUOA and a large fibroid at the base of the septum, it was deemed imperative to do the hysteroscopic myomectomy under laparoscopic observation of the uterine fundus to avoid uterine perforation and other complications. Figure 5a, 5b, and 5c illustrate the steps of resecting a large fibroid embedded at the base of the septum in another patient (patient # 8). Operative laparoscopy for endometriosis, pelvic adhesions or tubal factors was performed if such pathologies were encountered (Mitwally et al., 2009). At the conclusion of the procedure, a paediatric Foley’s catheter (size 8 French) was placed inside the endometrial cavity and its balloon was filled with 3 cc of normal saline. The catheter was removed after 7 days. We did not use any prophylactic antibiotics (Abuzeid O et al., 2018). Any intra-operative, immediate postoperative or late complications were reported to determine the safety of the operative procedure. A 6-week course of oral oestrogen was started 2 days after surgery with a progestogen added during the last 10 days of the oestrogen course. All patients underwent a TV 3D US with SIH 8 weeks after surgery for evaluation of the endometrial cavity.

**Figure 5a:**
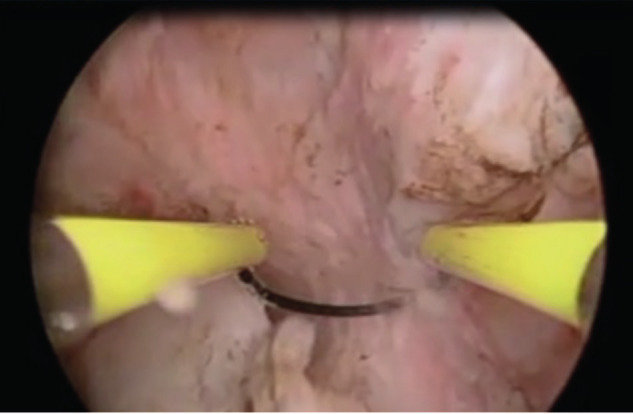
This picture illustrates hysteroscopic division of a complete septate uterus (patient # 8) using a straight resectoscope.

**Figure 5b:**
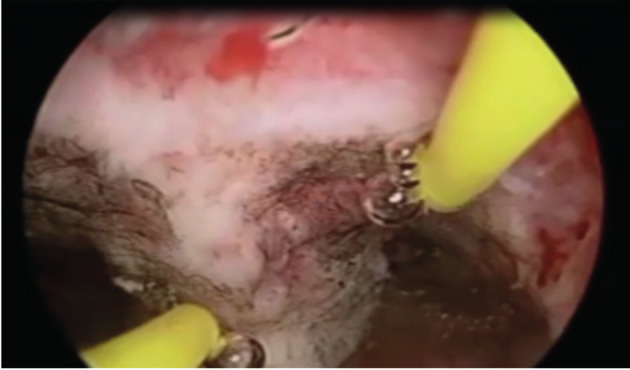
This picture illustrates hysteroscopic resection of a large fundal uterine fibroid that was embedded at the base of a complete uterine septum (patient # 8) using a right angle resectoscope loop.

**Figure 5c:**
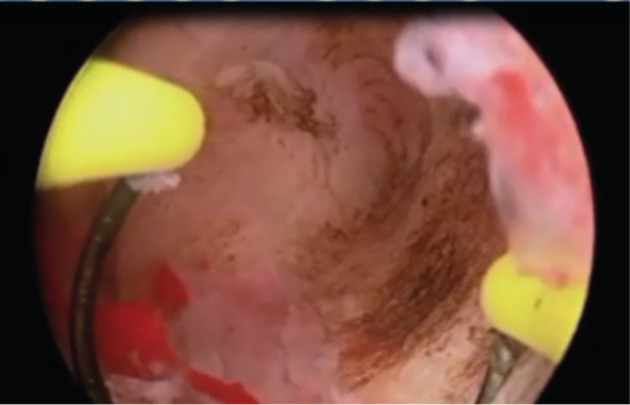
This picture illustrates the uterine fundus in patient # 8 at the conclusion of the procedure.

After surgery, depending on the underlying aetiology, patients were offered one of the following options to achieve pregnancy; spontaneous conception (SC) or controlled ovarian stimulation (COS) with gonadotropin injections with or without intrauterine insemination (IUI), or In-vitro fertilisation and embryo transfer (IVF-ET). In patients with associated ovulatory disorder, clomiphene citrate or an aromatase inhibitor was used for ovulation induction in the first instance. Patients who conceived were followed up to determine the pregnancy outcome. The efficacy of the operative procedure was determined based on the findings on postoperative TV 3D US with SIH and by reproductive outcomes.

## Results

Table I summarizes the background details. The mean age was 35.2 ± 3.9 years (range 31-43). Five patients had primary infertility (45.5%), while 6 patients had secondary infertility (55.5%). Five patients had a history of miscarriage (45.5%) and 2 patients had a history of RPL (18.2%). Male factors, ovulatory disorders, tubal factors and endometriosis accounted for 63.6%, 18.2%, 63.6% and 36.4% respectively. However, endometriosis was found in 4 out of the 7 patients who had a laparoscopy done (57.1%). Five patients underwent failed IVF-ET treatment prior to surgery 45.5%. Table II summarizes the data on fibroid numbers, location, type and size on TV 2D US. It also shows that in 2 patients no fibroid was detected on TV 2D US.

**Table I:** Background details of the patients.

Patient #	Age (years)	BMI Kg/m 2	Infertility Type	Infertility Duration (years)	Male Factor	Ovulatory Disorder	Tubal Factor	Endometriosis	FSH mIU/mL	AMH ng/ml	Hx of miscarriage	Number of Miscarriages	Laparoscopy	Hx IVF failure
1	33	30.0	Primary	5	Yes	No	No	N/A	8.5	4.37	No	N/A	No	Yes
2	34	28.3	Primary	1	Yes	No	Yes	Yes	8.0	2.32	No	N/A	Yes	No
3	34	25.6	Secondary	3.5	Yes	No	Yes	N/A	6.1	----	Yes	1	No	No
4	34	18.3	Primary	5	Yes	No	Yes	Yes	3.1	0.89	No	N/A	Yes	Yes
5	43	21.9	Secondary	5	No	No	Yes	N/A	17.1	----	Yes	3	No	Yes
6	34	34.0	Primary	1	Yes	No	Yes	No	6.2	----	No	N/A	Yes	No
7	36	24.6	Secondary	2	No	No	Yes	Yes	7.7	6.58	Yes	1	Yes	Yes
8	42	27.1	Secondary	1	No	No	No	N/A	13.1	2.09	Yes	2	No	No
9	31	43.1	Primary	2.5	No	No	Yes	Yes	6.9	----	No	N/A	Yes	Yes
10	35	46.6	Secondary	15	Yes	Yes	No	No	7.8	----	Yes	1	Yes	No
11	31	40.8	Primary	3	Yes	Yes	No	No	7	1.35	No	N/A	Yes	No

**Table II:** Data on fibroids on TV 2D US.

Patient #	Fibroid detected	Fibroid number	Fibroid location and (number)	Fibroid type	Fibroid size
1	Yes	3	Anterior (2) Posterior (1)	Intramural/subserous Intramural/subserous	7mm, 8mm 7mm
2	Yes	2	Posterior (1) Fundal (1)	Submucosal, intramural/subserous	11mm 11mm
3	No	N/A	N/A	N/A	N/A
4	Yes	2	Anterior (2) Posterior (1)	Intramural Intramural	16mm 8mm
5	Yes	2	Fundal (1) Fundal (1)	Submucosal/intramural Submucosal/intramural	10mm 9mm
6	Yes	2	Anterior (1) Anterior (1)	Intramural Intramural	10mm 9mm
7	Yes	7	Anterior (1) Posterior (1) Posterior (1) Fundal (1) Fundal (1) Fundal (1) Fundal (1)	Subserous Intramural/subserous Intramural/subserous Submucosal/intramural Intramural Intramural/subserous Intramural/subserous	15mm 12mm 13mm 10mm 17mm 20mm 24mm
8	Yes	1	Fundal (1)	Intramural/subserous	25mm
9	No	N/A	N/A	N/A	N/A
10	Yes	2	Posterior (1) Fundal (1)	Intramural/subserous Intramural/subserous	18mm 25mm

**Figure 6:**
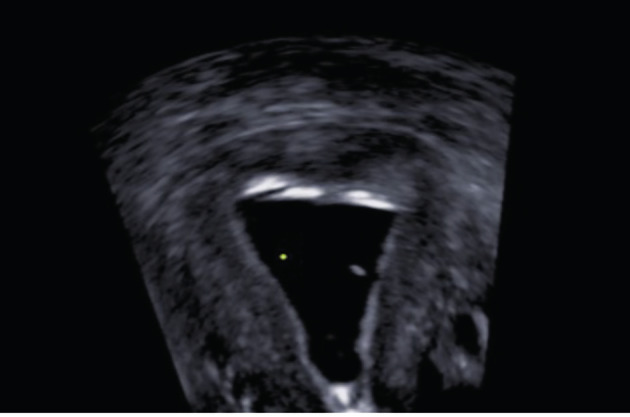
A normal postoperative TV 3D US with SIH eight weeks after concomitant hysteroscopic division of uterine septum and hysteroscopic myomectomy of a fundal fibroid embedded at the base of the septum.

Table III illustrates the radiological findings on preoperative TV 3D US with and without SIH, on hysteroscopy and postoperative TV 3D US with SIH. One patient had SU (9.1%) and the remaining 10 patients had PSU (90.9%). Six patients had PSUOA and the other 4 patients had PSUAA. In patients with PSU the mean IILFM on hysteroscopy was 15.4 ± 5.4 mm (range 12-30). The mean size of the fundal fibroid that was found embedded at the base of the septum was 12.5 ± 4.5 mm (range 5-20). Postoperative SIH was normal in 8 out the 11 patients who had the procedure done (72.7%) [Figure 6]. In 3 patients SIH was not normal. In 2 patients there were filmy adhesions and in one patient there was a piece of the fibroid still present at the fundal region. All 3 patients had a second hysteroscopic correction and subsequent SIH was normal. There was no report of any intraoperative, immediate, or late postoperative complications in this series. Pathology reports confirmed benign leiomyoma in all patients. Table IV illustrates pregnancy outcomes after surgery. In analysing the data in table IV; two patients who were >40 years of age, were excluded because of the effect of reproductive age on reproductive outcome. Seven patients conceived (77.8%), 6 patients delivered (66.7%), and one patient had a miscarriage at 10 weeks gestation (miscarriage rate of 14.3%). In addition, table IV illustrates the method of conception. Three patients conceived through IVF-ET, 2 spontaneously and one after COS and natural intercourse (NI). Three of 5 patients who failed IVF-ET treatment prior to surgery were able to conceive after surgery, one spontaneously and 2 with IVF-ET (Table I, Table IV).

**Table III:** Findings on preoperative TV 3D US, on hysteroscopy and postoperative TV 3D US with SIH.

Patient	PSUOA on TV 3D US	PSUAA on TV 3D US	CUS on TV 3D US	Type of uterine anomaly on hysteroscopy	IILFM on Hysteroscopy (mm)	Size of fibroid embedded in the septum (mm)	Postoperative TV 3D US with SIH
1	Yes	No	No	PSUOA	13mm	5mm	Normal
2	Yes	No	No	PSUOA	12mm	15mm	Normal
3	Yes	No	No	PSUOA	12mm	10mm	Small filing defect
4	Yes	No	No	PSUOA	13mm	10mm	Band of filmy scar tissue
5	No	Yes	No	PSUAA	13mm	10mm	Normal
6	No	Yes	No	PSUAA	13mm	15mm	Normal
7	No	Yes	No	PSUAA	20mm	10mm	Band of filmy scar tissue
8	No	No	Yes	CUS	30mm	20mm	Normal
9	No	Yes	No	PSUAA	15mm	10mm	Normal
10	Yes	No	No	PSUOA	15mm	15mm	Normal
11	Yes	No	No	PSUOA	13mm	20mm	Normal

Patients # 3, # 4 and # 7 had a second hysteroscopic correction of residual pathology and subsequent TV 3D US with SIH were normal.

**Table IV:** Post-operative reproductive outcome.

Patient	Pregnant	Miscarriage	Delivered	Method of conception	Mode of delivery	Gestational age at delivery	Obstetric complications
1	Yes	No	Yes	IVF-ET	Vaginal	37 weeks	None
2	Yes	No	Yes	SC	CS*	36 weeks	None
3	Yes	No	Yes	IVF-ET	CS	38 weeks	Cervical shorting / no cerclage
4	No	N/A	N/A	N/A	N/A	N/A	N/A
5	No	N/A	N/A	N/A	N/A	N/A	N/A
6	Yes	No	Yes	COS-NI**	CS	39 weeks	Pre-eclampsia
7	Yes	No	Yes	SC	CS	37 weeks	None
8	No	N/A	N/A	N/A	N/A	N/A	N/A
9	Yes	No	Yes	IVF-ET	CS	33 weeks	Preterm labor
10	Yes	Yes	No	IVF-ET	N/A	N/A	N/A
11	No	N/A	N/A	N/A	N/A	N/A	N/A

*Cesarean Section (CS) - **Natural Intercourse (NI)

## Discussion

To our knowledge this is the first report of a case series on the diagnosis and management of a concomitant uterine septum and uterine fibroids embedded at the base of the septum in the fundal region. A careful search of the English literature revealed 2 manuscripts and 2 abstracts on this topic (Caliskan et al., 2008; Iwase et al., (2013); Abuzeid et al., 2017a; Abuzeid et al., 2017b). Javid et al., (2013) reported on the need for 2 sessions to manage a concomitant uterine septum and a fundal submucosal/intramural/subserous fibroid. Since only a few case reports were found in the English literature on such an association, the assumption is that such combined pathology must be either very rare or under-reported. The fact, that we identified 11 cases of such concomitant pathology on hysteroscopy within 4 years indicates the rarity of such an association is due to under-reporting. A previous case series revealed the difficulty encountered during the diagnosis of uterine septa in the presence of uterine fibroids (Abdelaziz et al., 2014).

Uterine fibroids are common in females. In fact, one in four females have uterine fibroids (Baird et al., 2003). In addition, the most recent data suggests that the incidence of uterine septa is estimated to be 3.9% in infertile patients (Saravelos et al., 2008). TV 3D US with or without SIH can predict the presence of PSU (Jurkovic et al., 1997). However, the prevalence of PSU among infertility patients based on TV 3D US may be underestimated (Abuzeid et al., 2015; Abuzeid and Abuzeid 2014). In addition, TV 2D and 3D US can easily detect the presence of any uterine fibroid and determine its type and location (Mavrelos et al., 2011). However, previous investigators suggested that TV 3D US may not be accurate in the diagnosis of a uterine septum in the presence of uterine fibroids (Kupesic 2001).

Therefore, we believe that reproductive surgeons and gynaecologists should be familiar with this scenario prior to performing a hysteroscopic division of a uterine septum. This is especially the case, if there is evidence of uterine fibroids somewhere else in the uterus. Surgeons should also be aware that operative hysteroscopy is the most accurate method for the diagnosis of this combined pathology.

In this case series we described in detail the technique of hysteroscopic management of concomitant uterine septum/significant arcuate uterine anomaly and fibroids embedded in the base of the uterine anomaly in the fundal region. We demonstrate that in experienced hands, the procedure is safe and effective, provided that the surgeon has an excellent view throughout the procedure with adequate distension of the endometrial cavity. Additionally, a careful watch for any fluid deficit of non-electrolyte solution to avoid dilutional hyponatremia and its sequelae and other complications of fluid overload is imperative. Furthermore, care should be taken to avoid uterine perforation or thinning of the uterine fundus, which may result in uterine rupture during pregnancy or labour. We suggest that in difficult cases, with a large fibroid, as in case #11, a combined hysteroscopy and laparoscopy is warranted to ensure safety during the hysteroscopic resection of the large myoma at the base of the septum.

In this report we demonstrate the safety of a hysteroscopic correction of both pathologies at the same setting. This was based on the absence of immediate and late postoperative complications, and on the lack of any incidence of uterine rupture during pregnancy and delivery. In addition, the efficacy of this procedure is illustrated by the findings of normal postoperative SIH with no incidence in intrauterine scar tissue in 72.7% of patients, and in the remaining 27.3% after a second operative hysteroscopy. Furthermore, the efficacy of this procedure is illustrated by an impressive reproductive outcome. In fact, such efficacy may be further improved if postoperative evaluation is performed by direct visualisation of the endometrial cavity by hysteroscopy, in association with TV 3D US with SIH. This may allow for the detection and correction of any small pathology that can escape the indirect examination of the uterine cavity. In addition, since we reported that five patients underwent failed IVF-ET treatment prior to surgery (45.5%), and that three of these patients were able to conceive after surgery, one should further underline the importance of considering a hysteroscopic assessment of the uterine cavity in all patients before any IVF-ET procedure (Abuzeid et al., 2015; Abuzeid and Abuzeid, 2014).

## Conclusion

In experienced hands, a hysteroscopic myomectomy of a leiomyoma (small or large) embedded at the base of a uterine septum can be safely performed at the same setting of hysteroscopic division of the uterine anomaly. We also believe that the detection and surgical removal of such leiomyoma may improve the reproductive potential in such patients.
